# Dengue virus genomic variation associated with mosquito adaptation defines the pattern of viral non-coding RNAs and fitness in human cells

**DOI:** 10.1371/journal.ppat.1006265

**Published:** 2017-03-06

**Authors:** Claudia V. Filomatori, Juan M. Carballeda, Sergio M. Villordo, Sebastian Aguirre, Horacio M. Pallarés, Ana M. Maestre, Irma Sánchez-Vargas, Carol D. Blair, Cintia Fabri, Maria A. Morales, Ana Fernandez-Sesma, Andrea V. Gamarnik

**Affiliations:** 1 Fundación Instituto Leloir-CONICET, Avenida Patricias Argentinas 435, Buenos Aires, Argentina; 2 Department of Microbiology, Icahn School of Medicine at Mount Sinai, New York, New York, United States of America; 3 Arthropod-borne and Infectious Diseases Laboratory, Department of Microbiology, Immunology and Pathology, Colorado State University, Fort Collins, Colorado, USA; 4 Departamento Investigación, Instituto Nacional de Enfermedades Virales Humanas "Dr. Julio I. Maiztegui", ANLIS, Pergamino, Argentina; Institut Pasteur, FRANCE

## Abstract

The *Flavivirus* genus includes a large number of medically relevant pathogens that cycle between humans and arthropods. This host alternation imposes a selective pressure on the viral population. Here, we found that dengue virus, the most important viral human pathogen transmitted by insects, evolved a mechanism to differentially regulate the production of viral non-coding RNAs in mosquitos and humans, with a significant impact on viral fitness in each host. Flavivirus infections accumulate non-coding RNAs derived from the viral 3’UTRs (known as sfRNAs), relevant in viral pathogenesis and immune evasion. We found that dengue virus host adaptation leads to the accumulation of different species of sfRNAs in vertebrate and invertebrate cells. This process does not depend on differences in the host machinery; but it was found to be dependent on the selection of specific mutations in the viral 3’UTR. Dissecting the viral population and studying phenotypes of cloned variants, the molecular determinants for the switch in the sfRNA pattern during host change were mapped to a single RNA structure. Point mutations selected in mosquito cells were sufficient to change the pattern of sfRNAs, induce higher type I interferon responses and reduce viral fitness in human cells, explaining the rapid clearance of certain viral variants after host change. In addition, using epidemic and pre-epidemic Zika viruses, similar patterns of sfRNAs were observed in mosquito and human infected cells, but they were different from those observed during dengue virus infections, indicating that distinct selective pressures act on the 3’UTR of these closely related viruses. In summary, we present a novel mechanism by which dengue virus evolved an RNA structure that is under strong selective pressure in the two hosts, as regulator of non-coding RNA accumulation and viral fitness. This work provides new ideas about the impact of host adaptation on the variability and evolution of flavivirus 3’UTRs with possible implications in virulence and viral transmission.

## Author summary

Flaviviruses constitute the most important and diverse group of arthropod-transmitted viruses, including relevant human pathogens such as dengue, Zika, yellow fever, and West Nile viruses. The natural alternation of these viruses between vertebrate and invertebrate hosts imposes a selective pressure on the viral population, with potential epidemiological implications. However, the selective forces and mechanisms that act on the viral RNA during host adaptation are largely unknown. Using dengue virus, we found that viral replication in mosquito or human cells leads to the accumulation of different patterns of viral non-coding RNAs that differently regulate viral fitness in each host. Carrying out this process requires changes of the structure of the viral 3’UTR that is under strong selective pressure in the two hosts. Our findings provide the first link between flavivirus host adaptation, fitness, and the production of viral non-coding RNAs, and support a model in which opposite selective pressures in the two hosts drive flavivirus 3’UTR evolution.

## Introduction

Flaviviruses include a large number of emerging and re-emerging human pathogens that are mainly transmitted by arthropods, including dengue (DENV), Zika (ZIKV), yellow fever (YFV) and West Nile (WNV) viruses. Dengue is the most prevalent arthropod-borne viral disease around the world. It is endemic in more than 100 countries, with about 390 million infections each year [1]. In 2016, Latin America faced their worst dengue and Zika epidemics, without effective vaccines or antivirals to control infections. There are four antigenic and genetically distinct DENV serotypes (DENV1 to 4), each comprises multiple genotypes, which include distinct lineages, strains, or clades [2,3].

DENV epidemiological dynamics has the complexity of co-circulation of different viruses in endemic and hyper-endemic areas. Genetic variations between serotypes, genotypes and lineages are important determinants for differential viral fitness, virulence and epidemic potential [4–7]. Global genotype replacement events have been observed in different context; for instance, during the early 1990s, DENV2 from Southeast Asia displaced the American DENV2 in the Americas, resulting in more severe clinical outcomes in several Latin American countries [8]. Local DENV genotype and strain displacements also have been extensively documented [9–13]. In this regard, genotype displacements were observed in Sri Lanka (for review see [14]), in which sequential displacements of different DENV3 coincided with abrupt increases in severe dengue cases [7,15,16]. The factors that lead to DENV genetic variability and to viral replacements in nature are not well understood. However, a complex network of host-virus interactions, together with environmental factors, could account for the transmission of certain viral variants and not others.

One source of DENV genetic diversity is the natural alternation between invertebrate and vertebrate hosts, which imposes different selective pressures on the viral population [17–19]. Recent studies demonstrated that DENV2 adaptation to mosquito or human cells is associated with sequence diversification of viral 3’UTRs. Sequence analysis of DENV populations obtained from adult mosquitos or mosquito cells showed the selection of different variants with mutations that mapped in the 3’UTR of the viral genome that were cleared after host switch to human cells [17]. The source of the selective pressure and the mechanisms that explain the positive and negative selection of viral variants during host adaptation are still largely unknown. It has been hypothesized that sequence variations in the 3’UTR acquired during host adaptation were associated with the generation of viral-derived non-coding RNAs, known as subgenomic flavivirus RNAs (sfRNA) [20,21].

The sfRNAs are products of incomplete viral genome degradation by the host exonuclease Xrn1 in humans and mosquitos (for review see [20,22,23]). This exonuclease degrades the viral RNA progressively from the 5’ to the 3’ direction, and stalls when it encounters stable RNA structures, described as Xrn1 resistant RNAs (xrRNAs) present at the flavivirus 3’UTRs [24,25] [26]. The process renders RNA decay intermediates of 300 to 500 nucleotides of genomic 3’ ends that accumulate to high levels in flavivirus infected cells [24,27–30]. A number of recent studies have provided information about important functions of these sfRNAs, including evasion of cellular antiviral responses [24,31–36] [37]. Interestingly, sequence variability at the viral 3’UTRs was recently correlated with DENV epidemiological fitness and with accumulation of different sfRNA levels [38]. In this regard, a role of sfRNAs in binding TRIM25 and limiting RIGI dependent induction of type I interferon (IFN) was described [38].

These previous observations raise a number of questions: (1) Why do different DENV isolates generate distinct levels of sfRNAs? (2) Is the genetic variability observed in the viral 3’UTR during host adaptation linked to the production of viral non-coding RNAs? (3) Are the structural determinants for sfRNA accumulation similar in mosquito and human cells? To address these questions, we analyzed the accumulation of DENV non-coding RNAs in infected cells during the process of host adaptation. We found that viruses grown in mosquito or human cells generate different patterns of sfRNAs and these patterns rapidly change upon host switch. This process was not due to different properties of the host RNA decay machinery; but instead was attributed to specific mutations selected in each host. RNA structural analysis using viral variants positively selected in mosquito cells revealed that mutations in a single stem loop are the unique determinants for accumulation of short species of sfRNAs (sfRNA3 and sfRNA4) during infection. Interestingly, competition experiments indicated that viruses that generate sfRNA3 and sfRNA4 (uniquely produced by mosquito-adapted viruses) displayed low fitness in human cells and were quickly outcompeted by viruses that generate the long sfRNA1. Our studies explain how genetic variability at the DENV 3’UTR, generated during host adaptation, modulates the generation of specific patterns of viral non-coding RNAs linked to different fitness in each host. In addition, a comparative analysis of sfRNA accumulation during DENV and ZIKV infections indicated that their 3’UTRs are under different selective pressures.

## Results

### Different patterns of sfRNAs are generated by host adapted DENV

**Fig 1:**
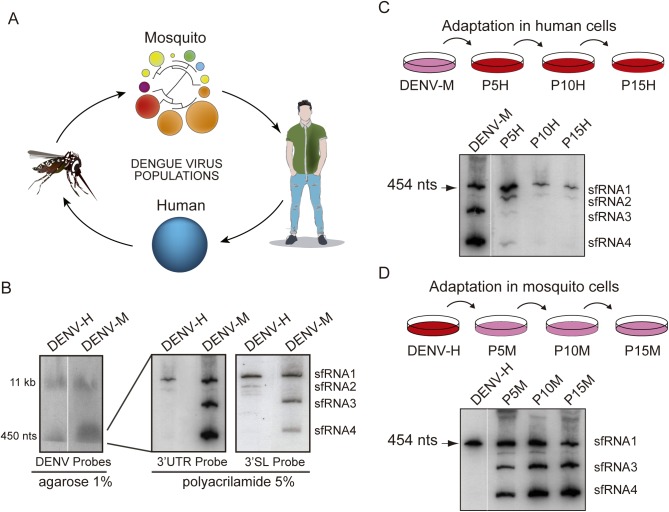
Different patterns of sfRNA produced during dengue virus host adaptation (**A**) Schematic representation of DENV 3’UTR diversification during host switch. Variants in viral populations obtained in mosquito or human cells are represented by circles. The size and color of circles represent the frequency and number of mutations of variants, respectively. The distance between variants is also shown by a fan dendrogram. (**B**) Genomic and subgenomic DENV RNAs produced in infections using viruses generated in human (DENV-H) or mosquito (DENV-M) cells. Cells were infected at MOI of 1 with DENV-H or DENV-M. Northern blots using a mix of radiolabeled probes that recognize the viral 3’UTR, capsid and NS5 (left), only the 3’UTR (middle) or only the 3’SL (right) are shown. The species of sfRNAs detected are indicated on the right. (**C**) Northern blot showing sfRNAs produced during the process of DENV adaptation to human cells. Specific probes complementary to the viral 3’UTR were used to hybridize RNA isolated from A549 cells infected with the indicated viruses. P5H, P10H and P15H indicate viral stocks passaged 5, 10 and 15 times in human cells, respectively. (**D**) Northern blot showing sfRNAs produced during the process of DENV adaptation to mosquito cells. Specific probes complementary to the viral 3’UTR were used to hybridize RNA isolated from C6/36 cells infected with the indicated viruses.

We have recently reported a dynamic process of positive and negative selection of different DENV variants during mosquito or human adaptation [17] (Fig 1A). Because a hot spot for these mutations was found in the viral 3’UTR within an RNA sequence possibly involved in sfRNA formation, we hypothesized that host specific selection of different variants is a mechanism to modulate sfRNA accumulation. To examine this possibility, production of sfRNAs in cells infected with viruses grown in human or mosquito cells was analyzed. Extracts of infected cells were subjected to Northern blot hybridization using a uniformly radiolabeled RNA probe complementary to the complete DENV 3’UTR. In 1% agarose/formaldehyde gels, genomic RNA with the expected size was observed together with an abundant subgenomic viral RNA in cells infected with the adapted viruses (Fig 1B, left). To examine this with more detail, samples were subjected to denaturing 5% polyacrylamide/urea gels. In these conditions, the human-adapted virus (DENV-H) generated mainly one sfRNA molecule (sfRNA1) and a low amount of a second RNA (sfRNA2). In contrast, the mosquito-adapted virus (DENV-M) gave rise to sfRNA1 along with two additional species (sfRNA3 and sfRNA4) (Fig 1B, middle). The non-coding RNAs observed shared the same 3’ end sequence because they were detected by a probe that hybridized only with the 3’SL sequence (Fig 1B, right). These results indicate that DENV grown in different host cells produce different species of sfRNAs.

To study the mechanism by which mosquito/human adapted DENV populations produce different sfRNAs, we examined the process of host adaptation. Viral stocks produced in each host were subjected to host switch and then passaged 15 consecutive times. Cell extracts at passage 5, 10 and 15 (P5, P10 and P15) were used for Northern blot analysis (Fig 1C and 1D). Interestingly, a change in the pattern of sfRNAs was directly associated with the adaptation process in both hosts. During human adaptation, a switch in the sfRNA profile was observed, from three main sfRNA species to a single sfRNA (sfRNA1, Fig 1C). The inverse process was observed in mosquito C6/36 cells, in which after 5 passages the three sfRNAs (sfRNA1, sfRNA3 and sfRNA4) were accumulated (Fig 1D). To extend these observations, viral adaptation was also performed in mosquito U4.4 cells. In this case, at passage 8, sfRNA1, sfRNA3 and sfRNA4 were observed (S1 Fig).

Together, these experiments indicate that DENV host adaptation has a direct impact on the generation and accumulation of distinct species of sfRNAs.

### Defining the nature of the sfRNAs produced in mosquito and human infected cells

**Fig 2:**
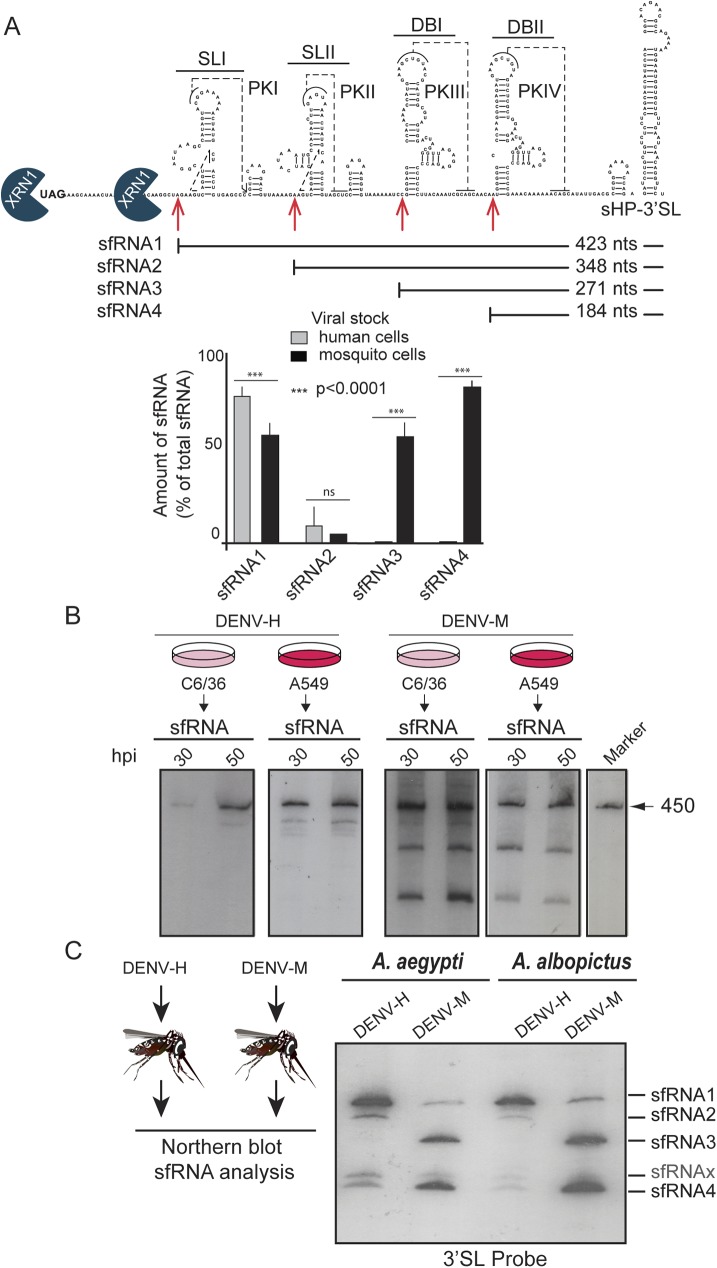
Mapping the sfRNAs generated in DENV infected human and mosquito cells. (**A**) Secondary structure of DENV 3’UTR indicating the location and size of sfRNAs identified by sequencing analysis. Below, plots representing relative amounts of sfRNA species produced using human or mosquito infected cells. The amount of each sfRNA was estimated by ImageJ-quantitation and expressed as the mean +/- SD of the relative percentage from total sfRNAs (n = 3). (**B**) Northern blot hybridization using specific probes complementary to the viral 3’UTR employing RNA extracted at 30 and 50 hpi from C6/36 or A549 cells infected with either DENV-M or DENV-H stocks, as indicated. (**C**) DENV sfRNAs produced in infected *Ae*. *albopictus* and *Ae*. *aegypi* mosquitos. Northern blot hybridization using RNA extracted from mosquitos infected with DENV-M or DENV-H. A probe complementary to the viral 3’SL was used for detection.

To understand the significance of the change of the patterns of sfRNAs, we sequenced the non-coding viral RNAs produced in DENV infected cells. To this end, purified monophosphorylated RNA molecules were circularized by ligation, subjected to reverse transcription, amplification, cloning and sequencing analysis. The 5’-3’ junctions were defined in each case (Fig 2A). The sequencing data indicate that sfRNA1, sfRNA2, sfRNA3 and sfRNA4 were 423, 348, 271 and 184 nucleotides long, respectively. Mapping these RNA molecules on the DENV 3’UTR indicated that sfRNA1 and sfRNA2 are the products of halting RNA degradation at the stem-loops (SLI and SLII, respectively), and sfRNA3 and sfRNA4 are the result of stalling degradation just upstream of each of the dumbbell structures (DBI and DBII, respectively) (Fig 2A). This indicates that SLI efficiently blocks the Xrn-1 activity in viruses grown in human cells, resulting in the accumulation of mainly sfRNA1 (90%). In contrast, in infections with mosquito adapted viruses, SLI does not efficiently restrict Xrn-1 degradation, and restriction at the DB structures is responsible for sfRNA3 and sfRNA4 accumulation (Fig 2A, bottom panel). This observation raises questions about possible reasons for these differences: Is the RNA decay machinery in mosquito and human cells differently affected by viral RNA structures? Is the sequence diversity of the viral population, with variants carrying different deletions within the 3’UTR in mosquito-adapted viruses, responsible for distinct sfRNA sizes?

To evaluate these possibilities, first we examined the production of sfRNAs in mosquito and human cells using viruses produced in both cell types in parallel. Viruses obtained from human cells (DENV-H) were used to infect both mosquito and human cells; and viruses obtained from mosquito cells (DENV-M) were also used to infect the two cell types (Fig 2B). We analyzed the sfRNAs at 30 and 50 h post-infection in the four cell extracts. The DENV-H generated mainly sfRNA1, both in C6/36 and in A549 cells (Fig 2B). On the other hand, the DENV-M produced the same pattern of sfRNA1, sfRNA3 and sfRNA4 in the two cell types. The sfRNAs produced by both viruses were the same in both cell types at 30 and 50 h post-infection, indicating that the observed patterns were not due to dissimilar infection kinetics of DENV-H and DENV-M. These results indicate that the specific sfRNA patterns observed are defined by determinants in the viral RNA and they are not a consequence of different host properties of the RNA decay machinery.

To extend our studies and evaluate possible host specific properties, we infected *Aedes albopictus* or *Aedes aegypti* adult mosquitos with DENV stocks generated in human or mosquito cells. An evident differential pattern of sfRNAs was observed by northern blot analysis using mosquito extracts (Fig 2C). *Ae*. *albopictus* and *Ae*. *aegypti* mosquitos infected with viruses obtained from human cells accumulated mainly sfRNA1. In contrast, infections with stocks generated in mosquito cells mainly accumulated sfRNA3 and sfRNA 4 (Fig 2C), confirming that host adapted viruses also generate qualitatively different patterns of sfRNA in whole infected mosquitos.

### xrRNA2 structure is the key regulator for changes in sfRNA accumulation during DENV host switch

**Fig 3:**
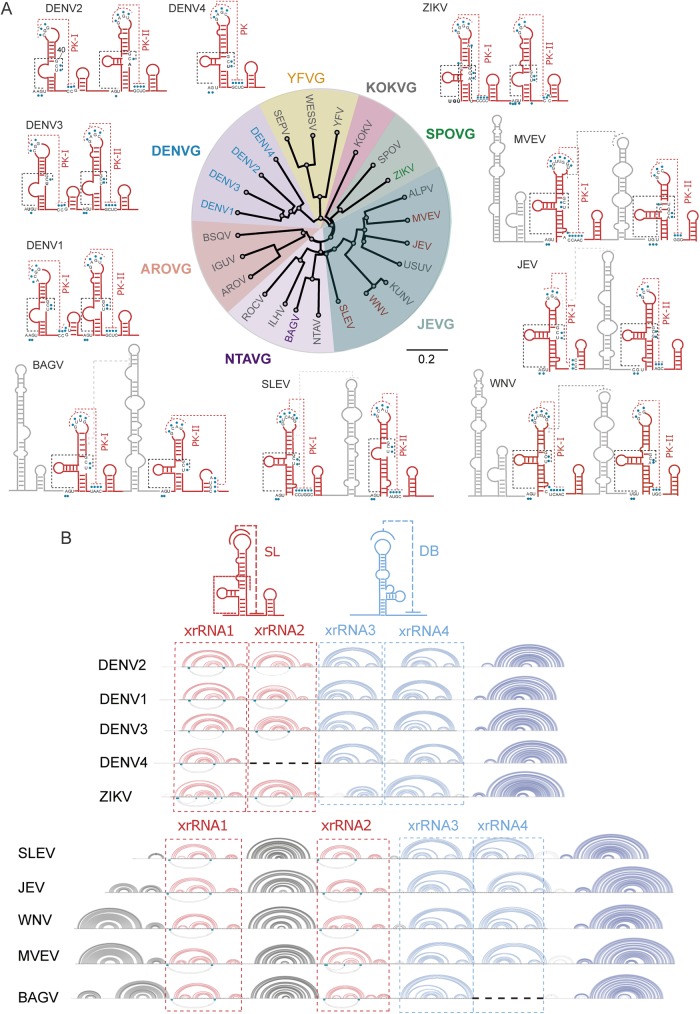
Comparative analysis of predicted RNA structures of the 3’UTR of mosquito-borne flaviviruses (MBFV). (**A**) Conserved stem loops of selected viruses from each subgroup of MBFV are shown in red. Predicted H-type pseudoknots are indicated with red dashed lines and pseudoknots including nucleotides present in the three-way junction are shown in black dashed lines. Information obtained from crystallographic studies using MVEV and ZIKV RNAs was included [25,39]. Group-specific RNA structures are indicated in grey. The distance tree was drawn using the neighbor joining method of all complete genome sequences for each virus available in GenBank. (**B**) Representation of the complete 3’UTR structure of different MBFVs showing the conserved elements involved in Xrn1 stalling. Conserved stem loop (SL) and dumbbell (DB) structures are shown in red and blue, respectively. Arc plots of RNA structures corresponding to the conserved xrRNA1, xrRNA2, xrRNA3 and xrRNA4 are shown for DENV1 to 4, ZIKV, WNV, MVEV, SLEV and BAGV. The conserved 3’SL structure is shown in dark blue.

Duplicated SL and DB elements that maintain conserved structural blocks and sequences have been observed in the 3’UTR of most mosquito borne flaviviruses (MBFV) (Fig 3A and 3B). These elements include H-type pseudoknot (PK) interactions that stabilize the RNA structures. In addition, X-ray crystallography of MVEV RNA gave evidence for an unpredicted PK interaction between 5’ end sequences and nucleotides present in the three-way junction, providing stability to the RNA structure for halting the Xrn1 activity [25]. This interaction appears to be conserved among different flaviviruses including co-variations [20], supporting the significance of these contacts (Fig 3A, black dashed lines). More recently, crystallographic studies using Zika virus RNA revealed additional unpredicted contacts [39].

Members of the Japanese encephalitis (JEV), Kokobera (KOKV) and Ntaya (NTAV) groups contain unrelated RNA elements preceding the conserved SL structures (Fig 3A, grey solid lines). Because these RNA elements were originally named SLs, it was unclear when the conserved SLs involved in sfRNA formation from different flaviviruses were compared. In order to analyze molecular determinants for sfRNA formation in DENV, and correlate them with those observed in other flaviviruses, it was important to use a common nomenclature. In this regard, it was recently proposed the functionally descriptive names of xrRNA1, xrRNA2, xrRNA3 and xrRNA4 for the conserved RNA structures involved in Xrn1 stalling in MBFV 3’UTRs [26]. For clarity, the corresponding structures of different flaviviruses are depicted in Fig 3B.

In order to define the genetic determinants of viral non-coding RNA accumulation during DENV infection and the cause of the changes in the patterns of sfRNAs observed during host adaptation, we dissected viral populations and analyzed properties of individual variants.

**Fig 4:**
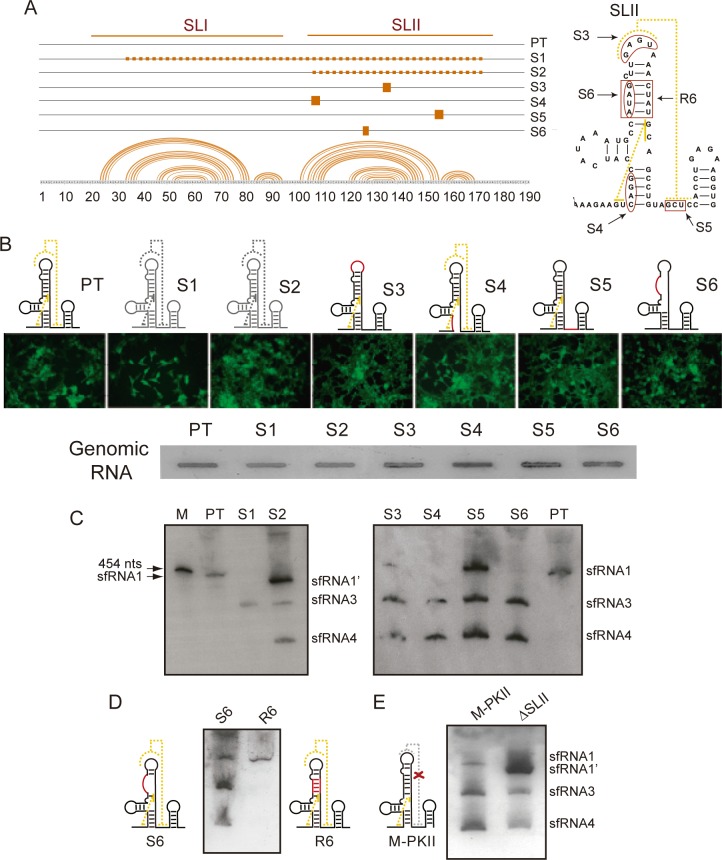
Dissecting the molecular determinants for sfRNA accumulation during DENV host change. (**A**) Schematic representation of mosquito-selected viral variants with mutations and deletions within the viral 3’UTR. DENV variants were found with deletions, variant S1 and S2, or point mutations variants S3 to S6. The location of the mutations is indicated. (**B**) Replication of parental (PT) and recombinant viruses carrying the mutations identified. Immunofluorescence of cells infected PT and the mutants S1 to S6 as indicated at the top. Viral RNA detection using specific radiolabeled probes complementary to capsid and NS5 coding sequences is shown at 72hpi of A549 cells (bottom). (**C**) Northern blot hybridization for sfRNA detection using transfected cells with each DENV mutant as indicated on the top. **(D)** The structure of xrRNA2 is necessary for accumulation of short sfRNA species. Schematic representation of changes in the xrRNA2 structure of mutant S6 and restored R6, and Northern blot shown the accumulated sfRNAs in each case. **(E)** Point mutations within xrRNA2 are sufficient for the switch of sfRNA pattern produced during DENV infections. Schematic representation of mutation abrogating PK formation (M-PKII) is shown on the left and Northern blot comparing the accumulation of sfRNAs detected in infected cells with M-PKII or a virus with a complete deletion of xrRNA2 (ΔSLII).

Alignment of viral genomes from mosquito adaptations showed variants with large deletions of 63 and 132 nucleotides, including sequences of xrRNA1 and xrRNA2, and different variants with specific mutations within the SL of xrRNA2 (Fig 4A and [17]). We hypothesized that viral variants with large deletions, including the SL structures, were responsible for generating the shorter sfRNA2, sfRNA3, and sfRNA4 and that the pattern of sfRNAs observed in infected cells and whole mosquitos was a mixture of RNA products from different viruses present in the population. To examine this possibility, we generated recombinant viral RNAs by introducing the individual mutations into the viral 3’UTR. Six full genome RNAs were generated with defined mutations: S1 with a deletion of 132 nucleotides, S2 with a deletion of 63 and S3 to S6 with point mutations (Fig 4A).

In vitro transcribed RNAs from the parental (PT) and mutated infectious clones were transfected into A549 cells and immunofluorescence was performed at 3-days post-transfection. Except for the mutant with the largest deletion (S1), which displayed a delay in replication, the other mutants (S2 to S6) replicated efficiently (Fig 4B). Extracts from transfected cells were directly used to evaluate accumulation of genomic RNA and production of sfRNAs, to avoid selection of revertant viruses. The mutants produced similar levels of viral genomic RNA at 48 h post-transfection (Fig 4B). Interestingly, each mutant accumulated defined species of sfRNAs. The mutant S1 produced only sfRNA3, indicating that, upon deletion of part of xrRNA1 and xrRNA2, Xrn-1 stopped at the first DB. Surprisingly, the mutants that contained defined mutations or deletions only within SLII generated sfRNA1, sfRNA3 and sfRNA4 or mainly sfRNA3 and sfRNA4 (S4 and S6). This unexpected result indicates that even point mutations within SLII were sufficient for Xrn-1 degradation through xrRNA1 and xrRNA2, supporting the idea that accumulation of sfRNA3 and sfRNA4 is directly modulated by xrRNA2 structure, regardless of the presence of an intact xrRNA1. In addition, the data indicate that the two DB structures efficiently halt the Xrn1. The results suggest that the different species of sfRNAs observed are not a mixture of sfRNAs from a variety of viral variants present in the population, instead they are generated by defined mutations in the SL of xrRNA2. The data support the idea that the switch in the pattern of sfRNAs in mosquito-adapted viruses is caused by specific changes in the xrRNA2 structure.

Finding that alterations in the SL structure of xrRNA2 are responsible for reducing sfRNA1 formation seemed counterintuitive because sfRNA1 is associated with the halting activity of xrRNA1. To rule out the possibility that mosquito-adaptive mutations within xrRNA2 could have an unpredicted influence on the 3’UTR structure, we used the variant S6 and reconstituted the SL structure with compensatory mutations. In the S6 viral RNA context, secondary mutations restoring the stem of xrRNA2 were introduced (mutant R6, Fig 4D). RNAs corresponding to S6 and R6 were used comparatively to evaluate the production of sfRNAs. The results show that reconstitution of SLII of xrRNA2, by introduction of mutations into S6, restored stalling of Xrn1 at xrRNA1, rendering a WT-like sfRNA1 (Fig 4D). These results indicate that the xrRNA2 structure is necessary for DENV sfRNA1 accumulation.

To further confirm a functional coupling of xrRNA1 and xrRNA2 structures, we analyzed the impact of point mutations or deletion of xrRNA2 on xrRNA1 activity. For this, two viral RNAs were used: one with the complete deletion of SLII (ΔSL) and the other carrying a specific mutation that impaired PK formation (M-PK). Both viruses produce sfRNA3 and sfRNA4 (Fig 4E). Interestingly, the M-PK mutation was sufficient to impair xrRNA1 and xrRNA2 functions and accumulated mainly the shorter sfRNAs. These results indicate that the halting activity of the duplicated RNA structures in DENV (xrRNA1 and xrRNA2) are strongly coupled, providing a mechanism for the regulation of Xrn1 activity on the viral 3’UTR.

Together, the results provide compelling evidence for a key function of xrRNA2 in modulating the switch in the pattern of sfRNAs produced after host change. In addition, because the SLII structure is both a hot spot for mosquito adaptive mutations and the determinant for the regulation of sfRNA production, the data suggest that DENV 3’UTR evolution is driven by distinct requirements of sfRNAs in different hosts.

### Accumulation of specific species of sfRNAs has an impact on DENV fitness in human cells

**Fig 5:**
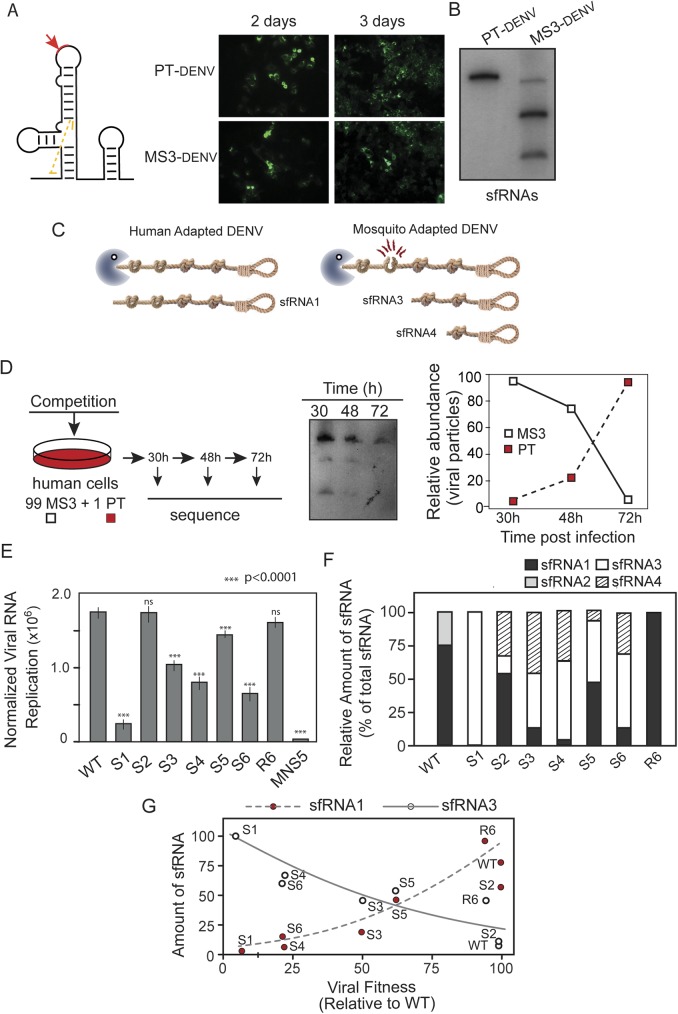
Fitness in human cells and sfRNA production of mosquito adapted DENV (**A**) Replication of parental (PT) and mutant (MS3) viruses in A549 cells monitored by immunofluorescence at 2 and 3-day post transfection. (**B**) Accumulation of sfRNAs in A549 cells transfected with PT or MS3 viral RNAs detected by Northern blot at 3-days post transfection. (**C**) Schematic representation of the key role of xrRNA2 for differential sfRNA accumulation in mosquito or human adapted viruses. Rope knots represent the xrRNA structures that impair Xrn1 movement and the gallows represents the terminal 3’SL structure. (**D**) Growth competition experiments highlight the fitness disadvantage of mosquito selected variant in human cells. A549 cells were infected with a mixture of PT: MS3 at a 1:99 ratio. At 30, 48 and 72 hpi, cells were collected for viral RNA purification,sequencing and sfRNA analysis. Relative abundance of each virus is represented in the plot shown on the right. **(E)** Viral RNA replication levels of WT and mosquito selected variants are shown together with a replication impaired control (mutant in the polymerase NS5, MNS5) in A549 cells. Mutations were incorporated in a replication and propagation competent reporter DENV carrying a luciferase gene. Normalized luciferase levels are shown in a logarithmic scale at 48h post-transfection. The values are the mean +/-SD, n = 3. **(F)** Quantitation of sfRNAs accumulation in A549 cells transfected with mutant RNAs. The values are the mean relative accumulation estimated with ImageJ Program from three independent experiments. **(G)** Plot of the mean relative amounts of different sfRNAs, shown in F, versus the replication capacity measured using the reporter virus shown in E.

We demonstrated that DENV host change from mosquito to human cells leads to an abrupt change in the composition of the viral population and in the pattern of sfRNAs ([17] and Fig 1) and the determinants for this change were specifically mapped in xrRNA2. Thus, we propose a link between a negative selection of mosquito adapted viruses in human cells and the requirements for specific species of sfRNAs in distinct hosts. In order to evaluate this possibility, we first analyzed the viral fitness of host-adapted viruses. Our initial studies showed that replication of most of the cloned viral variants with mutations in the 3’UTR selected in mosquitos, replicated similarly to variants selected in human cells when analyzed by immunofluorescence in A549 cells (Fig 4B). To better assess viral fitness, we performed growth competition experiments with viruses with defined genetic backgrounds. A virus with a mutation that was repeatedly acquired during replication in mosquito cells (carrying mutation S3), named virus MS3, and the parental virus (PT) were used. Viral stocks were generated, sequenced and titrated by plaque assays. Infectivity in A549 cells was analyzed by immunofluorescence as a function of time, and the generation of sfRNAs was examined by northern blot at day 3 post-infection (Fig 5A and 5B). During replication of virus MS3, sfRNA3 and sfRNA4 were the main sfRNAs accumulated, while the PT virus produced sfRNA1, confirming that point mutations were sufficient to switch the pattern of sfRNAs (Fig 5C). Competition experiments were carried out by mixing the two viruses and the frequency of each in the population was assessed by sequencing cloned 3’UTRs at different time points. In conditions in which a ratio of 99:1 MS3 to PT were used, the frequency of MS3 changed from 99% to less than 1% in only 72 h (Fig 5D), indicating a great replication advantage of the virus that accumulated sfRNA1. The relative fitness, calculated as previously described [40], indicates that MS3 replicated 3-fold less well than the PT virus.

To extend the link between sfRNA accumulation and DENV fitness in human cells, a sensitive assay to assess viral replication was used. Reporter viruses that carry a luciferase gene were generated for the variants selected in mosquito cells. Replication kinetics of the reporter viruses with mutations S1 to S6 and R6 was evaluated together with the parental virus (PT). Viral translation was similar for all the variants, but RNA replication measured between 24 and 48 h showed significant differences compared to the parental virus (Fig 5E). Virus S1 showed the greatest reduction in replication, also detected by IF (Fig 4B). The variants S3, S4, S5 and S6 showed between 2 and 4 fold-reduction in viral RNA amplification. In addition, variant R6, with mutations in both strands of SLII that reconstituted the RNA structure, showed PT levels of replication. Next, we examined how efficiently the viruses replicated with respect to the specific sfRNA species accumulated in each case. To estimate the relative amounts of sfRNAs (sfRNAx/total sfRNA x100), we used northern blots from three independent experiments. The mean relative amounts of different sfRNAs (Fig 5F) was plotted against the replication capacity measured using the reporter virus (Fig 5G). An evident positive correlation between viral replication capacity and the ability of the virus to accumulate sfRNA1 was observed. In contrast, accumulation of sfRNA3 showed an inverse correlation with viral fitness. These results indicate that viruses with genetic variations associated with mosquito adaptation having low levels of sfRNA1 display reduced fitness in human cells.

**Fig 6:**
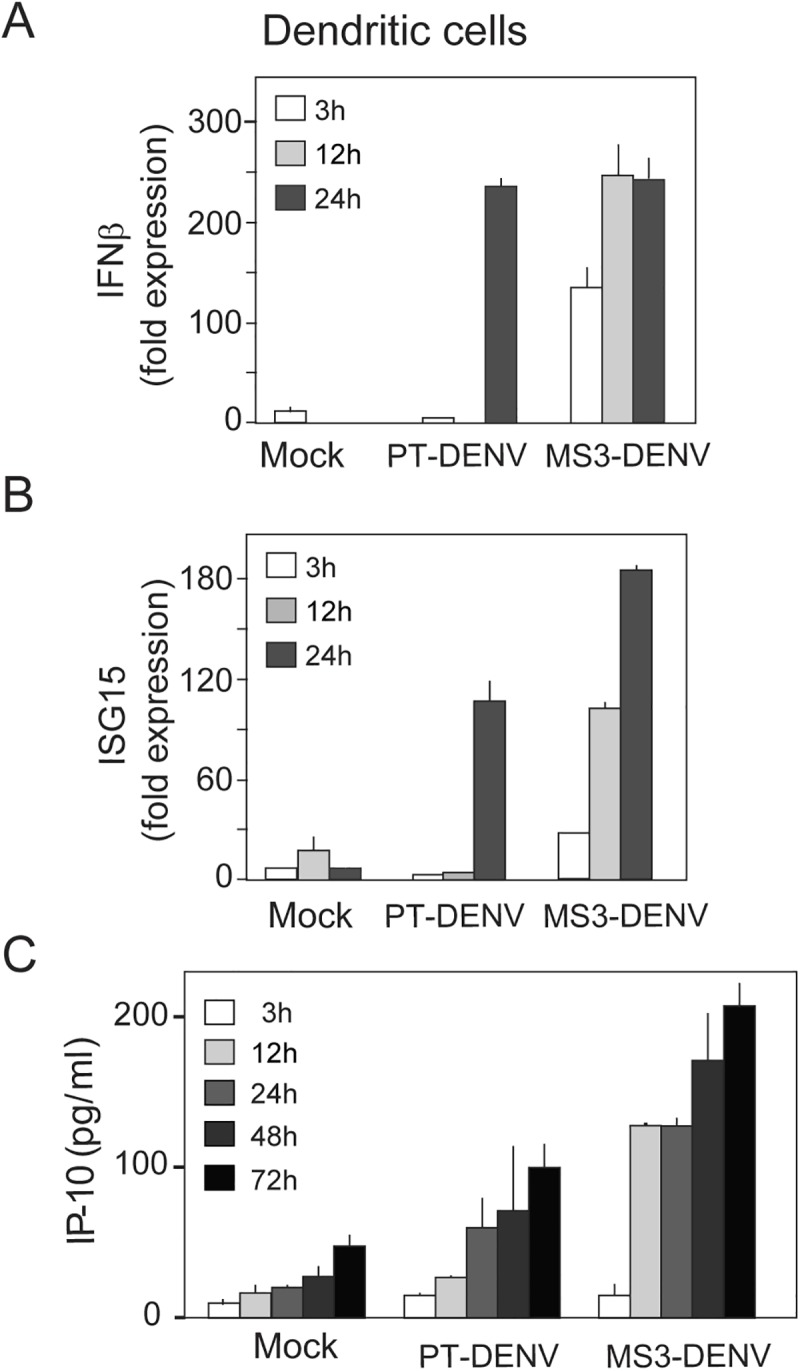
Mosquito adapted DENV variant exhibit an exacerbated antiviral response in human cells. Time course analysis of antiviral response in dendritic cells (DCs). DCs of three independent donors were infected with PT or MS3 virus, and at 3, 12, and 24 hpi IFNβ (**A**) or ISG15 (**B**) mRNA were determined by real time PCR. (**C**) The levels of IP-10 in the supernatant of infected cells was assessed by ELISA at 3, 12, 24, 48 and 72 hpi. Data are the means and standard deviations of three replicates from a representative donor.

The lack of sfRNA1 in WNV infections has been associated with defects in counteracting the IFN response [34], and DENV natural isolates that accumulate low levels of sfRNAs showed a defect in controlling RIG-I activation [38]. Thus, to explore the possible cause of the reduced viral replication of viruses adapted to mosquitos in human cells, we evaluated the antiviral response in monocyte-derived human dendritic cells (DCs) infected with DENV carrying defined mutations associated with mosquito adaptation. DCs are natural targets of DENV infection that secrete and respond to type I interferon (IFN), producing co-stimulatory molecules and cytokines [41]. A time course analysis after infection indicated that virus MS3 induces earlier and higher levels of type I IFN as compared to the parental virus (Fig 6A). This observation was reproduced with DCs from three independent donors. In addition, measurement of the IFN stimulated genes (ISGs) IP10 and ISG15 support a stronger antiviral response during infection with the MS3 virus (Fig 6B and 6C). These results indicate that a mosquito adapted virus induces a stronger antiviral response in human cells, supporting the relevance of sfRNA1 in counteracting the host type I IFN response, as previously reported, and explaining the clearance of mosquito selected viruses during DENV replication in human cells.

### Unique properties of sfRNAs in ZIKV infections in mosquito and human cells

**Fig 7:**
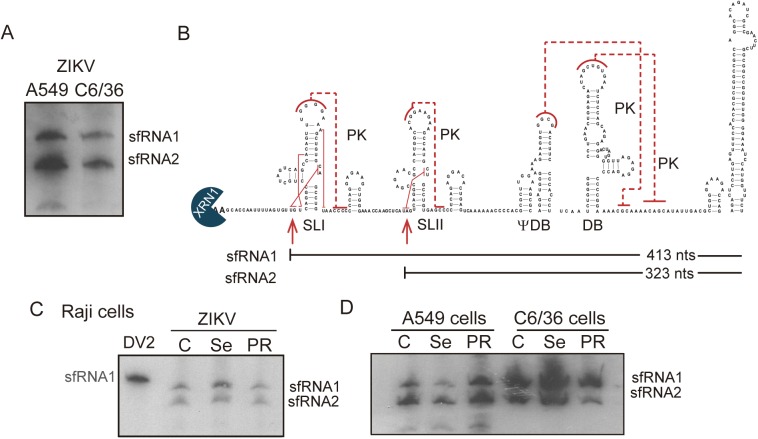
Production of sfRNAs in Zika virus infections. (**A**) Northern blot hybridization showing the accumulation sfRNA in C6/36 and A549 cells infected with a Zika isolate from Argentina (INEVH). (**B**) RNA structure model predicted for the ZIKV 3’UTR. Conserved SLI, SLII, pseudo-dumbbell (ψ-DB) and DB structures are indicated. Pseudoknots are indicated with red lines. Location and size of the two sfRNAs identified in infected cells are shown (sfRNA1 and sfRNA2). (**C and D**) Northern blots showing the pattern of sfRNAs in Raji, A549 and C6/36 cells infected with ZIKV from different origins (Cambodia, Senegal and Puerto Rico) as indicated on the top.

We have observed that DENV adaptation to adult mosquitos or mosquito cells leads to genetic variability in the viral 3’UTR [17], which is associated with a reduced viral fitness and a stronger immune response in human cells (Figs 5 and 6). To test whether these trends are shared among other flaviviruses, we experimentally analyzed the pattern of sfRNAs in ZIKV infected cells. To this end, a virus isolated in Argentina (INEVH 71516) was used to infect mosquito and human cells. Cytoplasmic extracts of infected cells were subjected to Northern blot analysis employing specific ZIKV probes. The data revealed the accumulation of similar amounts of two species of non-coding RNAs (Fig 7A). Sequencing of purified RNAs indicated that the two sfRNAs were 413 and 323 nucleotides long, likely produced by Xrn1 stalling at xrRNA1 and xrRNA2, respectively (Fig 7B). RNA structure prediction and co-variation analysis of ZIKV 3’UTRs indicate the presence of conserved duplicated SL structures (xrRNA1 and xrRNA2). Interestingly, recent crystallographic studies identified unpredicted tertiary interactions within xrRNA1 [39] (Fig 7B). In addition, a peculiar pseudo-dumbbell (ψ-DB) and a DB structure were predicted based on co-variation studies (Fig 7B). The ψ-DB was predicted to form a PK with sequences present downstream of the DB, resembling the organization of RNA elements in YFV 3’UTR [21].

A recent report indicated that ZIKV genomes from epidemic and pre-epidemic isolates displayed sequence variations at the 3’UTRs, including predicted changes in xrRNA1 structure [42]. Thus, to evaluate whether ZIKV from Asia, Africa or America produce different patterns of sfRNAs, infections with viruses from different origins were performed in Raji cells. The infections showed accumulation of similar levels of sfRNA1 and sfRNA2 (Fig 7C, left panel). In addition, this pattern did not change when the viruses were grown in mosquito or human cells (Fig 7C, right panel). These results indicate that the sequence variations observed in the 3’UTR of ZIKV isolates do not significantly alter the quality of sfRNAs produced and suggest that ZIKV and DENV differently modulate the production of viral non-coding RNAs during host adaptation.

## Discussion

Here, we provide an explanation for the negative and positive selection of DENV variants after host switch. We found that the process of host alternation imposes a selective pressure on RNA structures present at the viral 3’UTR that regulate the accumulation of distinct species of viral non-coding RNAs. This mechanism requires structural changes within xrRNA2 and depends on a functional coupling of duplicated RNA elements. We demonstrated that mutations that were fixed in the viral population grown in mosquito cells were sufficient to impair xrRNA1 function, avoid sfRNA1 formation, limit the virus’s ability to counteract the antiviral response in human cells and reduce viral fitness, explaining the rapid clearance of mosquito-selected viral variants after host switch. We propose a model in which different requirements of viral non-coding RNAs in different hosts drive DENV 3’UTR evolution, providing a basis for genetic variations observed in natural isolates.

A growing body of evidence supports the relevance of the accumulation of sfRNAs during flavivirus infections, in epidemiological fitness and pathogenesis (for review see [23]). Our work adds layers of complexity to this phenomenon, revealing that the obligatory DENV cycling between vertebrate and invertebrate hosts exerts a strong pressure on the kind of sfRNAs produced during infection. In this regard, viruses that replicate in human cells generate mainly sfRNA1, while mosquito-adapted viruses accumulate sfRNA3 and sfRNA4. The determinants for production of different species of sfRNAs were found to be in the viral RNA and not in differences in the host RNA decay machinery. Importantly, we ruled out the assumption that sfRNA3 and sfRNA4 were generated by large deletions found in populations adapted to mosquito cells, since selected point mutations in recombinant viruses were sufficient to generate the short sfRNAs.

Work from different laboratories provided a great deal of information about possible mechanisms for sfRNA functions, including control of the antiviral response in human and insects [20,32,34–36,38,43]. Using DENV, we found that mosquito-selected variants induced a strong antiviral response in human DCs. In these experiments, earlier and higher levels of IFN-β IP-10 and ISG15 were detected as compared to human-adapted DENV variants (Fig 6). This observation could be due to a viral limitation in counteracting the antiviral response caused by low sfRNA1 levels, or, alternatively, by more efficient sensing of the shorter sfRNA species generated by mosquito-adapted viruses. In the case of WNV, the full length sfRNA1 has been specifically associated with counteracting the host antiviral response, and viruses carrying mutations designed to reduce sfRNA1 accumulation displayed attenuation of virulence and higher sensitivity to type I IFN [34]. In this regard, the direct correlation between DENV fitness in human cells with the ability to produce sfRNA1 also supports a function for this non-coding RNA in DENV infections in human cells (Fig 5). We conclude that mosquito-selected viruses carrying defined variations in the viral 3’UTR displayed a lower ability to overcome the human antiviral response.

The key function found for xrRNA2 in modulating the accumulation of sfRNAs during DENV infection was unexpected. We and others have previously proposed that the duplication of structural elements at the flavivirus 3’UTRs, specifically xrRNA1 and xrRNA2, function as a backup mechanism [17,20]. In fact, the opposite requirement of structural elements within xrRNA2 for DENV replication in mosquito and human cells that leads to sequence variability, but the maintenance of an intact xrRNA1, was recently interpreted as a mechanism to ensure sfRNA1 accumulation [21,36]. However, the experimental data obtained here with DENV infections indicate that xrRNA1 and xrRNA2 are heavily coupled, and point mutations in xrRNA2 drastically reduce sfRNA1 accumulation. Therefore, DENV evolved a mechanism to shift the sfRNA pattern by incorporating single nucleotide changes in one RNA structure. Structural details of how mutations in xrRNA2 impair Xrn1 halting at xrRNA1 are still unclear. In agreement with this observations, a coupling between xrRNA1 and xrRNA2 was previously described using WNV [29] [20] [26]. In that case, mutations in xrRNA2 also decrease the halting activity of xrRNA1. It is likely that the two RNA structures participate in higher order interactions by unpredicted RNA-RNA contacts or mediated by bridging proteins. Importantly, the data indicate that rather than having a redundant function, the duplicated RNA structures evolved different, but coordinated, roles.

RNA sequence and structural comparison of xrRNA1 and xrRNA2 from different MBFV suggest an early diversification of functions. Using LocARNA [44], which simultaneously folds and aligns RNA sequences, allowing the incorporation of structural constraints from biochemical probing and conservation analysis, indicate that xrRNA2s of different viruses of a MBFV group are more closely related than xrRNA1 and xrRNA2 of the same virus (S2 Fig). This observation provides evidence that RNA structure duplication took place before diversification of each group. In addition, the relative stabilities of xrRNA1 and xrRNA2 can explain the unique properties of sfRNA produced during replication of different flavivirus. In the case of WNV, a higher stability for xrRNA1 results in efficient halting of the exonuclease at this structure [24,29]. In the case of ZIKV, similar stabilities of xrRNA1 and xrRNA2 may explain the similar efficiencies in stalling the Xrn1, resulting in the accumulation of comparable amounts of sfRNA1 and sfRNA2 (Fig 7). It was particularly interesting to examine epidemic and pre-epidemic genotypes of ZIKV since a recent report suggested that nucleotide variations can alter xrRNA1 structure. However, comparisons of ZIKV isolates from Argentina, Puerto Rico, Cambodia, and Senegal, showed accumulation of similar sfRNA patterns. Moreover, this pattern was maintained when the virus was grown either in mosquito or human cells. These results obtained with ZIKV, together with the coupled activity of the xrRNAs observed using DENV, suggest that experimental data is necessary to interpret the impact of natural sequence variations on the ability of a virus to accumulate different patterns of sfRNAs.

Sequence alignments of DENV isolates indicate variations and covariations within xrRNA2 from different genotypes, but less variation was found in xrRNA1 (S3 Fig). This raises the question if mosquito adaptation contributes to this genetic variability observed in the 3’UTRs of natural DENVs. In previous studies using experimental infections in whole mosquitos, few mutations were identified in the viral 3’UTR [19]. This could be explained by the small amount of replicated copies of viral RNA obtained from mosquitos, and the time required for selection. Interestingly, growth competition experiments in *Ae*. *albopictus* indicated that viruses carrying mutations in the xrRNA2 structure displayed higher fitness than those with an intact RNA structure, and viral genome sequences from naturally infected mosquitos showed specific mutations and deletions in this RNA element (S3 Fig), supporting a replication advantage of variants with mutations within xrRNA2 in mosquitos. Here, we observed a rapid clearance of mosquito-selected DENV variants in human cells (Fig 5D). It is possible that mosquito selected viruses are maintained in human populations due to bottleneck processes during transmission. In this regard, viral isolates from patients were reported to accumulate different sfRNA species. For instance, a DENV2 isolate from China, obtained from a patient with dengue fever, was shown to accumulate three sfRNA species, similar to those observed here following mosquito adaptation [45], supporting efficient transmission of this virus. In addition, different ratios of sfRNA/genomic RNA were reported for DENV isolates from Puerto Rico, and the relative levels of sfRNA produced were correlated with viral epidemiological fitness [38]. We conclude that positive selection of mutations in the viral 3’UTR that provide fitness advantages in mosquitos can explain the variations observed in natural DENV isolates that generate reduced amounts of sfRNA1, and/or different kinds of sfRNAs, with implications in viral transmission between mosquitos and humans.

In summary, we provide a novel mechanism by which DENV evolved an RNA structure that confers opposite levels of fitness in mosquito and human cells, as a regulator of non-coding RNA accumulation during infection. In addition, this work provides new information about the origin and selective pressures that lead to flavivirus genetic variability at the 3’UTR, providing new insights for understanding strain displacements and viral spread.

## Materials and methods

### Ethics statement

Blood from healthy human donors were obtained from the New York Blood Center. These samples are anonymous blood bank donor samples that do not require IRB review.

### Viral transfections, infections and experimental host adaptation

DENV infections were performed using different cell lines as indicated in each case. Human lung cells (A549, ATCC, CCL-185) were grown at 37°C in D-MEM medium. Mosquito C6/36HT (*Aedes albopictus* cells ATCC, CRL-1660 adapted to grow at 33°C) and U4.4 (*Aedes albopictus* cells RRID:CVCL _Z820) were maintained in Leibovitz's L-15 Medium, supplemented with 10% fetal bovine serum, 100 U/ml penicillin, 100 μg/ml streptomycin, 0.3% tryptose phosphate, 0.02% glutamine, and 1% MEM non-essential amino acids solution. To obtain human and mosquito adapted viruses, in vitro transcribed DENV RNAs from full-length cDNA of serotype 2 pD2IC were transfected using lipofectamine in A549 or C6/36 cells respectively, viruses were harvested at 3 days (P1) and successive infections were performed in the same line using a multiplicity of infections of 1.

### Northern blots

For DENV genome and sfRNA detection, total RNA was obtained using Trizol reagent from infected cells at 72 hpi and separated on 1% agarose/2% formaldehyde or 5% polyacrylamide/7M urea gels. RNA was then transferred onto a nylon membrane (Hybond-N; GE Healthcare) using a semidry blotting apparatus and UV-crosslinked. Membrane was blocked for 1 h at 60°C in hybridization solution (50% formamide, 1mg/ml bovine serum albumin, 750 mM sodium chloride, 75 mM sodium citrate, 0.1 mg/ml herring sperm DNA, 1% sodium dodecyl sulfate, 1 mg/ml polyvinylpyrrolidone, 1 mg/ml ficoll). Uniformly ^32^P -labeled RNA probes were obtained by in vitro transcription using T7 RNA polymerase (Ambion) and purified by G-50 columns (GE Healthcare). RNA probes complementary to different regions of the DENV genome were designed and used, as described in each experiment. DENV probe: included an RNA complementary to the 5’UTR sequence (nucleotides 1–160) and RNAs directed to the coding regions of Capsid and NS5 (nucleotides 201–410; and nucleotides 1080–10250, respectively). The 3’UTR probe included two RNAs, one complementary to the complete 3’UTR (nucleotides 10269–10723) and the second complementary to the 5’ region of the 3’UTR (nucleotides 10269–10369). The 3’SL probe included one RNA complementary to this structure (nucleotides 10617–10723). Hybridization was performed by overnight incubation at 60°C. Blots were then washed twice with low stringency wash solution (300 mM sodium chloride, 3 mM sodium citrate, 0,1% sodium dodecyl sulfate) at 25°C, twice with medium stringency wash solution (30 mM sodium chloride, 0,3 mM sodium citrate, 0,1% sodium dodecyl sulfate) at 25°C and twice with the same solution at 42°C; and twice with high stringency wash solution (15 mM sodium chloride, 0,15 mM sodium citrate, 0,1% sodium dodecyl sulfate) at 68°C. Membranes were dried and hybridized RNAs were visualized by autoradiography or PhosphoImaging analysis. The amount of each sfRNA was relatively quantified by ImageJ program.

To obtain RNA from DENV infected mosquitos, New Orleans *Aedes albopictus* and Chetumal *Aedes aegyti* laboratory strains were reared from eggs and maintained as adults at 28°C, and 80% relative humidity with a photocycle of 12 h light:12 h dark, and given water and sugar until infection. Adult female mosquitoes 5 day post-emergence were intrathoracically (IT) inoculated with approximately 70 nl cell culture medium containing human or mosquito adapted DENV (200 PFU) using a Nanojet II (Drummond Scientific Company). Mosquitos were maintained for 14 days and used for RNA extractions using Trizol reagent.

### Mapping the sfRNAs

To identify the sfRNA species, Trizol-extracted RNA from infected C6/36 or A549 cells were used. The ends of 5’phosphorylated RNA molecules were end-ligated by an RNA ligase (Epicentre) and used as template for reverse transcription with primer AVG42 (GCTGTTTTTTGTTTCGGG). The cDNA molecules were then used as template for PCR reactions with different sets of internal primers. Products were gel purified and sequenced using Sanger method and the 5’-3’ end junction was identified.

### Phylogenetic and RNA secondary structure analysis

Phylogenetic trees were constructed using all available complete genomes of mosquito-borne flaviviruses group DENV1-4, SEPV, WESSV, YFV, KOKV, SPOV, ZIKV, ALPV, MVEV, JEV, USUV, KUNV, WNV, SLEV, NTAV, BAGV, ILHV, ROCV, AROV, IGUV and BSQV employing a neighbor-joining method. For RNA structure conservation analysis, representative viruses corresponding to mosquito-borne flaviviruses were used. The RNAfold package software was used to detect thermodynamically stable and evolutionarily conserved RNA secondary structures. To characterize possible tertiary interactions, complementary base pairs and co-variations, Biostring functions and the RNAaliduplex software were used together with predicted secondary structure models.

### Construction of recombinant DENVs

Mutations were introduced in the full-length cDNA of DENV 2 pD2/IC AflII, replacing the Afl*II*-Xba*I* fragment of the WT plasmid with the respective fragment derived from overlapping PCRs containing the desired mutations as previously described [46]. To generate S1 mutant, we used primers AVG64 (AAGCAGGAGTTCTGTGGTAGCGGCCGCTCCACCTGAGAAGGTGT) and AVG65 (ACACCTTCTCAGGTGGAGCGGCCGCTACCACAGAACTCCTGCTT); for S2, AVG1249 (cAAGGACGTTAAAAGCCACCTGAGAAGGTG) and AVG1250 (CACCTTCTCAGGTGGCTTTTAACGTCCTTG); for S3, AVG1362 (CCATAGCTTTTCGAAACTATGCAGCCTGTA) and AVG1363 (TAGTTTCGAAAAGCTATGGCATTTAT); for S4, AVG1372 (TGAGTAAACTATGCAGGGACTAGCTCCACCTGAGAAG) and AVG1373 (CTTCTCAGGTGGAGCTAGTCCCTGCATAGTTTACTCA); for S5, AVG1368 (CCATAGCTTGAGTAAACTATGCAGCCTGTACGAACACCTGAGAAGGTGT) and AVG1369 (GTTTACTCAAGCTATGGCATTTATGATGGCCTGCAATCTTTTAACGTCCTT); and for S6, AVG1374 (CCATAGCTTGAGTAAAGATAGCAGCCTGTAGCTCCA) and AVG1375 (TGGAGCTACAGGCTGCTATCTTTACTCAAGCTATGG). For reporter virus studies, the mutations were introduced in the context of the mDV-R replicon virus [47]. Transfected cells with WT or mutated full-length DENV RNA were used for immunofluorescence (IF) assays. A549 cells were grown in 35-mm-diameter tissue culture dishes containing a 1-cm2 coverslip inside. The coverslips were removed and directly used for IF analysis.

### Competition experiments

For competition experiments, viral stocks of S3 mutant (MS3) and the parental virus were obtained and titrated by plaque assays. A mixture of S3 and parental viral stocks (99:1; based on infectious viral titers) were used to infect A549 cells. The inoculum, and samples taken at 30, 48, and 72 hpi, were used for sequencing. RNA was extracted and RT PCR to amplify the complete 3’UTR with primers AVG458 (TAGAAAGCAAAACTAACATGAAAC) and AVG459 (AGAACCTGTTGATTCAACAGCAC). PCR products were ligated into pGEM-T Easy (Promega) vector and used to transform DH5α bacteria. For each time point, 24 clones were sequenced by Sanger method and ratio of MS3/PT virus were calculated.

### Dendritic cell infections and type I IFN response analysis

For infection of dendritic cells, monocyte-derived dendritic cells (DCs) were obtained by culturing CD14+ cells, isolated from blood of healthy donors and incubated in the presence of granulocyte-macrophage colony-stimulating factor (GM-CSF), interleukin-4 and human serum for five days. All infections were performed in DCs from at least three independent donors. Supernatant and cellular pellet were separated by centrifugation and total RNA from the cell pellets was trizol-extracted at different times post-infection and used for q-RT-PCR to extensively analyze the expression profile of the type I IFN response, using primers specific for human IFNβ and ISG15. IP-10 in the supernatant of the infected MDDCs was measured with ELISA from Millipore following manufacturer instructions.

### Zika virus subgenomic analysis

Clinical isolates from Argentina were obtained from the Instituto Maiztegui (INEVH), Pergamino, Buenos Aires. Viral stocks of Senegal (KX198134), Cambodia (KU955593), and Puerto Rico (PRVABC59) were used to infect Raji, A549 and C6/36 cells. At 60 hpi, total RNA was extracted and northern blot assays were performed as described above. 3’UTR complementary probes were obtained by T7 transcription and designed with primers AVG2004 (GCAACCAATTTAGTGTTGTCA) and AVG2005 GAAACCATGGATTTCCCCACACCGGC).

## Supporting information

S1 Fig DENV adaptation in U4.4 cells. A. Different patterns of sfRNA produced during DENV adaptation to U4.4 mosquito cells. Cells were infected at MOI of 1 with DENV-H. Viruses were passaged successively 8 times, as indicated on the top scheme. Northern blots, using specific probes complementary to the viral 3’UTR, were performed to analyze the accumulation of sfRNAs in U4.4 cell extracts after infection with each passage. B. Sequence of cloned variants present in the P8 population. The location of mutations within SLII structure are indicated.
(TIF)

S2 Fig Structure-based distance tree of xrRNA1 and xrRNA2. Representative sequences of DENV1 to 4, SLEV, WNV, JEV and ZIKV xrRNAs were aligned using LocARNA software [44]. Input RNA structure information from biochemical probing and conservation analysis was included. The color code shows in blue xrRNA1 and in red xrRNA2. The single structure of DENV4 was labeled in orange.
(TIF)

S3 Fig Sequence variations within the xrRNA1 and xrRNA2 structures from different genotypes of DENV2. Nucleotide changes with respect to a reference Asian genotype are highlighted in red. Phylogenetic tree was constructed by neighbor-joining method using Asiatic I 16681, (NC_001474), Asiatic II (NGC, M29095.1), Asian-American (CUB_115, AY702036.1), Cosmopolitan (TSV01, AY037116.1), American (IQT2913, AF100468.1), Sylvatic (African) (DakAr510, EF105381.1), Sylvatic (Asian) (P8-1407, EF105379.1) sequences. At the bottom, nucleotide sequence of DENV2 isolated from different *Aedes* mosquitos are shown. Nucleotide variations and deletions are indicated in red in the predicted RNA structure.
(TIF)
