# Supplementary figures and images for: Dengue virus genomic variation associated with mosquito adaptation defines the pattern of viral non-coding RNAs and fitness in human cells
Source: PLoS Pathog. 2017 Mar 6;13(3):e1006265. doi: 10.1371/journal.ppat.1006265 (PMC5354447; doi:10.1371/journal.ppat.1006265)

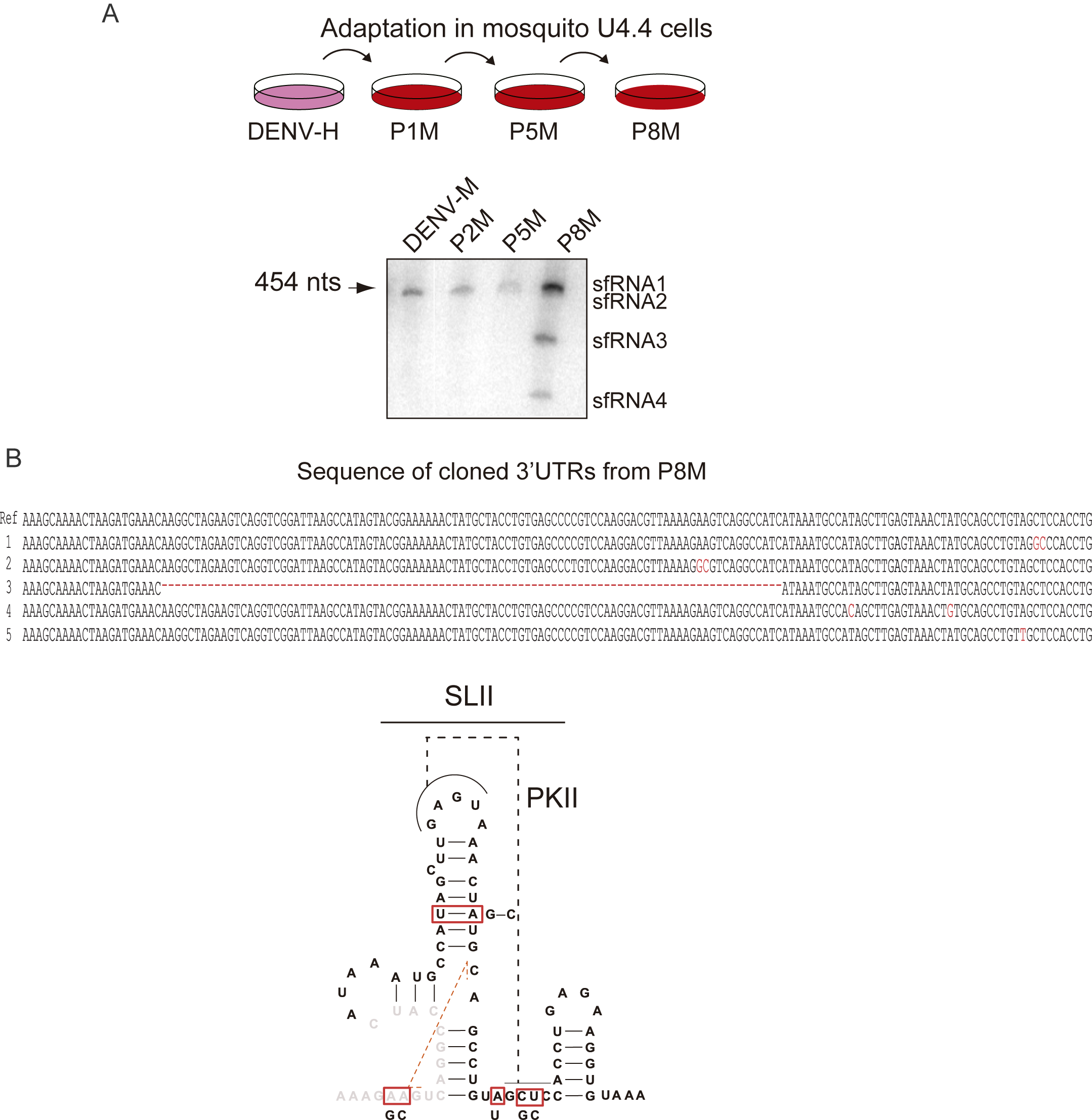

Supplement: S1 Fig — A. Different patterns of sfRNA produced during DENV adaptation to U4.4 mosquito cells. Cells were infected at MOI of 1 with DENV-H. Viruses were passaged successively 8 times, as indicated on the top scheme. Northern blots, using specific probes complementary to the viral 3’UTR, were performed to analyze the accumulation of sfRNAs in U4.4 cell extracts after infection with each passage. B. Sequence of cloned variants present in the P8 population. The location of mutations within SLII structure are indicated. (TIF) [file ppat.1006265.s001.tif]

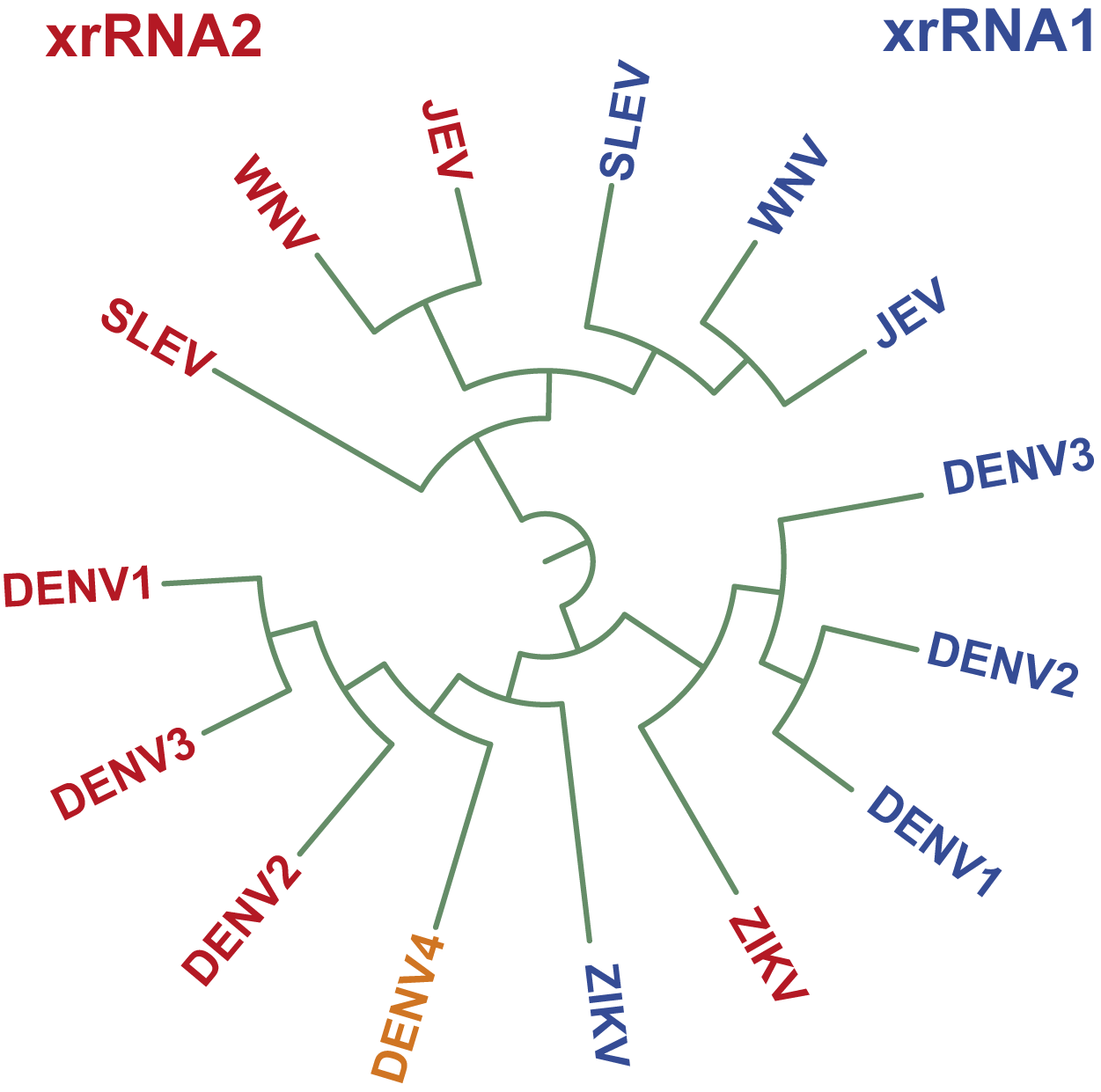

Supplement: S2 Fig — Representative sequences of DENV1 to 4, SLEV, WNV, JEV and ZIKV xrRNAs were aligned using LocARNA software [44]. Input RNA structure information from biochemical probing and conservation analysis was included. The color code shows in blue xrRNA1 and in red xrRNA2. The single structure of DENV4 was labeled in orange. (TIF) [file ppat.1006265.s002.tif]

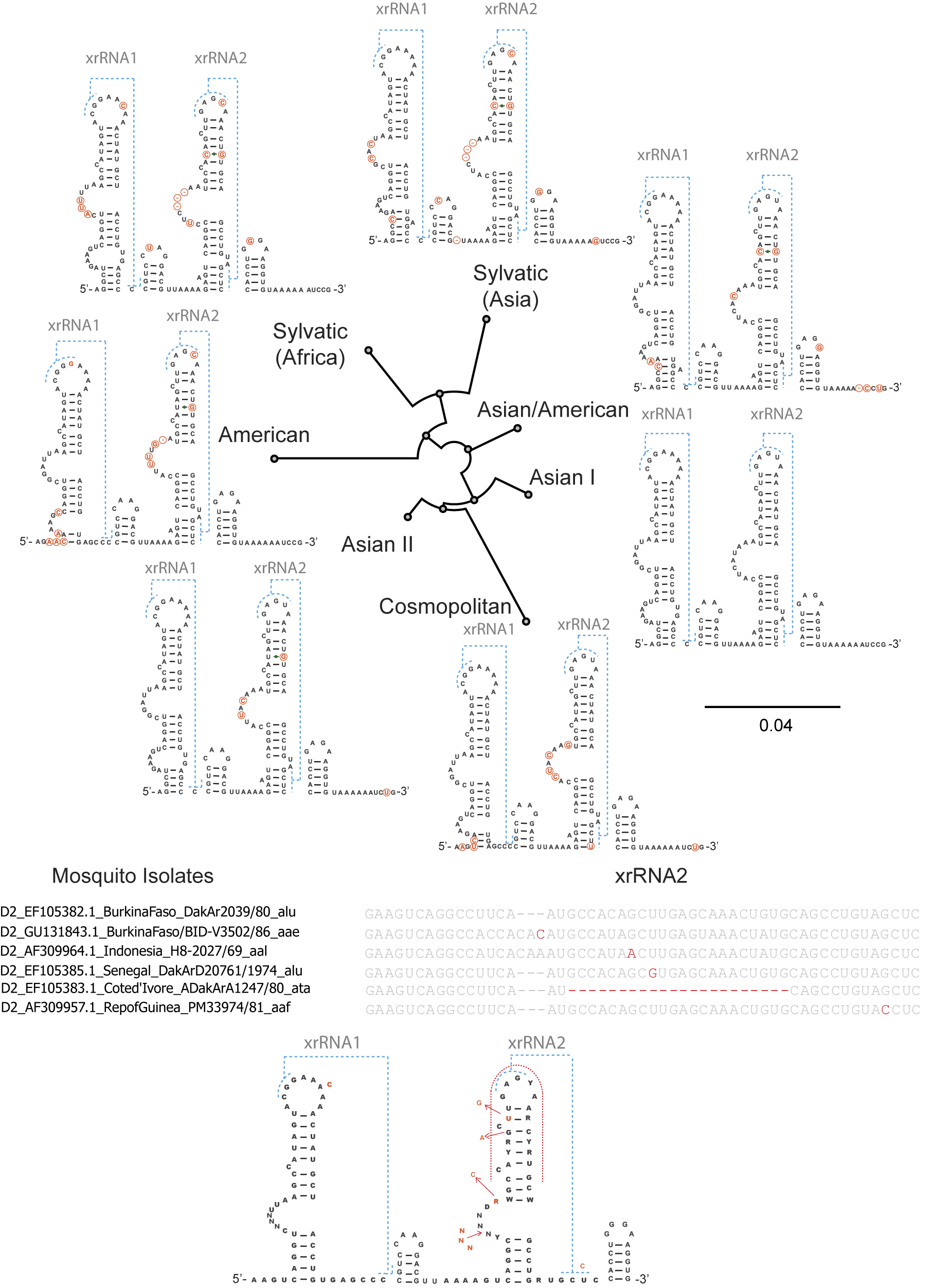

Supplement: S3 Fig — Nucleotide changes with respect to a reference Asian genotype are highlighted in red. Phylogenetic tree was constructed by neighbor-joining method using Asiatic I 16681, (NC_001474), Asiatic II (NGC, M29095.1), Asian-American (CUB_115, AY702036.1), Cosmopolitan (TSV01, AY037116.1), American (IQT2913, AF100468.1), Sylvatic (African) (DakAr510, EF105381.1), Sylvatic (Asian) (P8-1407, EF105379.1) sequences. At the bottom, nucleotide sequence of DENV2 isolated from different Aedes mosquitos are shown. Nucleotide variations and deletions are indicated in red in the predicted RNA structure. (TIF) [file ppat.1006265.s003.tif]
